# Optical Coherence Tomography Changes in Central Nervous System Inflammatory Demyelinating Diseases: A Longitudinal Retrospective Study

**DOI:** 10.7759/cureus.102978

**Published:** 2026-02-04

**Authors:** Duaa T Daradkeh, Mohammad A AL Shdaifat, Mutaz N Sarayrah, Anzor R Al Alwan, Walaa A Shatnawi, Ahmed Khatatbeh

**Affiliations:** 1 Ophthalmology, King Hussein Medical Center, Amman, JOR; 2 Ophthalmology, Royal Medical Services, Amman, JOR

**Keywords:** demyelinating disorder, multiple sclerosis, neurodegeneration, oct (optical coherence tomography), optic neuritis

## Abstract

Background

Optical coherence tomography (OCT) is considered an indispensable, non-invasive imaging modality that provides insights into neuroaxonal integrity in several retinal and optic nerve disorders by offering high-resolution, cross-sectional visualization of retinal and optic nerve head layers. With its capability of providing imaging of retinal microstructures, recently, OCT has been used in the assessment of central nervous system inflammatory demyelinating diseases (CNS-IDDs). These diseases, such as clinically isolated syndrome (CIS), relapsing-remitting multiple sclerosis (RRMS), secondary progressive multiple sclerosis (SPMS), neuromyelitis optica spectrum disorder (NMOSD), and myelin oligodendrocyte glycoprotein antibody-associated disease (MOGAD), are frequently associated with visual pathway impairment. Our purpose in this research is to evaluate longitudinal OCT changes and visual function across CNS-IDD subgroups.

Methods

This is a retrospective cohort study including 80 patients with CNS-IDDs (10 CIS, 30 RRMS, 20 SPMS, 10 NMOSD, 10 MOGAD) who underwent OCT at baseline, 3, 6, 9, and 12 months at the Ophthalmology department at King Hussein Medical Center. All patients received a detailed ophthalmic assessment, including measuring visual acuity (VA) using the Snellen chart under standard illumination, performing OCT to measure both ganglion cell-inner plexiform layer (GCIPL) and peripapillary retinal nerve fiber layer (pRNFL). The optic nerve head was assessed to determine the presence or absence of swelling using dilated fundoscopy and optic nerve head photos. Optic disc swelling was recorded as a categorical variable. Longitudinal and subgroup comparisons were analyzed using repeated-measures GLM and Bonferroni-adjusted post hoc tests.

Results

At baseline, CIS patients showed the most favorable parameters (VA 0.05 ± 0.008 LogMAR; GCIPL 85.06 ± 0.43 µm; pRNFL 97.00 ± 0.36 µm), while NMOSD exhibited the poorest outcomes. Over 12 months, all groups showed a continued decline of VA and thinning of GCIPL and pRNFL (p < 0.001). Optic disc swelling increased from 50.0% at baseline to 76.3% at 12 months, eventually affecting all SPMS, NMOSD, and MOGAD patients. Pairwise analyses confirmed significant subgroup differences, with NMOSD consistently demonstrating the worst structural and functional outcomes.

Statistical analysis revealed significant longitudinal reductions in VA, GCIPL, and pRNFL thickness across all subgroups (p < 0.001). Between-group comparisons showed significant differences for baseline and 12-month values (CIS vs NMOSD, p < 0.001; RRMS vs SPMS, p = 0.02; MOGAD vs SPMS, p = 0.04). Changes over time within each diagnostic category also reached statistical significance (all p < 0.001 by repeated-measures GLM).

Conclusion

OCT clearly showed a pattern of progressive neuroretinal degeneration across all CNS-IDD subgroups. The most significant changes were detected in NMOSD and SPMS, whereas CIS showed relative preservation, highlighting the potential role of OCT in monitoring disease course and differentiating phenotypes. Our findings emphasize the clinical value of OCT as a sensitive, non-invasive, reliable biomarker of visual pathway changes in CNS demyelinating diseases.

## Introduction

Central nervous system inflammatory demyelinating diseases (CNS-IDDs) cover a broad spectrum of immune-mediated disorders, including multiple sclerosis (MS), neuromyelitis optica spectrum disorder (NMOSD), and myelin oligodendrocyte glycoprotein antibody-associated disease (MOGAD). They are typically described as conditions presenting with visual pathway involvement, with optic neuritis (ON) representing one of the most common initial clinical manifestations. Optic nerve inflammation may occur in isolation or with other clinical manifestations in immune-mediated central diseases. It is characterized by subacute visual loss in one or both eyes, often associated with pain during eye movements and variable recovery of optic nerve function. Visual impairment often persists as a major source of disability in certain circumstances, making the monitoring of retinal structural changes pivotal in clinical practice [1-3].

MS is one of the most common CNS-IDDs, characterized by focal demyelinated plaques and inflammation within the central nervous system. ON in MS usually manifests with acute or sub-acute monocular visual loss, mainly affecting the central vision initially, progressing to affect peripheral vision. Simultaneous bilateral ON is rare in MS, compared to ON in NMOSD and MOGAD [4,5].

NMOSD with the aquaporin-4 (AQP4) autoantibody is associated with florid demyelination and inflammation, involving three or more contiguous spinal cord segments and the optic nerves. It is characterized by acute attacks of bilateral or rapidly sequential ON (leading to severe visual loss) or transverse myelitis with a typically relapsing course [4-6]. The visual loss accompanied by ON is more severe in NMSOD compared to MS, although individual ON attacks is indistinguishable initially from clinically isolated syndromes or those related to MS. However, bilateral or rapid sequential ON are more likely to be associated with an atypical form of immune-mediated central demyelinated disorders such as NMOSD or MOGAD [7-9]. Thus, early discrimination between NMOSD ON and MS ON is crucial to provide appropriate and timely management.

Patients with MOGAD are more likely to involve the optic nerve than the spinal cord, mostly in the picture of simultaneous bilateral ON affecting a longer segment of the optic nerve. The course of MOGAD is more likely to be monophasic, has fewer relapses, and is less likely to be associated with other autoimmune disorders [10,11].

Optical coherence tomography (OCT) has emerged as a useful, non-invasive imaging technology capable of quantifying retinal layers into separate thicknesses with high resolution [6,8,9]. In particular, thinning of the peripapillary retinal nerve fiber layer (pRNFL) and ganglion cell-inner plexiform layer (GCIPL) has been recognized as a marker of axonal and neuronal loss in CNS central demyelinating diseases. In addition to visual acuity (VA), OCT is considered a strong objective tool in assessing neurodegeneration, complementing the findings of magnetic resonance imaging (MRI) and clinical assessment [8,12-14].

The primary objective of this study was to evaluate 12‑month longitudinal changes in best‑corrected visual acuity (BCVA), GCIPL thickness, and pRNFL thickness across central nervous system inflammatory demyelinating disease (CNS‑IDD) subgroups. Secondary objectives were to compare the rates of structural and functional decline among disease subtypes and to analyze the frequency and progression of optic disc swelling using Frisén grading. We speculated that OCT would reveal distinct patterns of retinal neurodegeneration across subgroups, with more aggressive changes in NMOSD and progressive MS phenotypes compared with clinically isolated syndrome (CIS) and relapsing-remitting multiple sclerosis (RRMS).

## Materials and methods

Study design and setting

This was a longitudinal retrospective cohort study utilizing prospectively collected clinical and OCT data conducted at the neuro-ophthalmology clinic in King Hussein Medical Centre (KHMC), Amman, Jordan, a tertiary referral centre. It enrolled 80 patients diagnosed with CNS-IDDs between January 2023 and December 2024. Medical records of patients were reviewed during this period.

Study population and sampling technique

A consecutive sampling technique was used. All patients diagnosed with CNS-IDDs who attended the neuro-ophthalmology clinic during the study period and met the criteria were included in the study.

Study population

A total of 80 patients were enrolled and divided into 5 diagnostic groups based on international diagnostic criteria. These included RRMS (n = 30), CIS (n = 10), secondary progressive multiple sclerosis (SPMS; n = 20), NMOSD (n = 10), and MOGAD (n = 10).

Inclusion and exclusion criteria

Patients were included if they were aged ≥18 years at enrolment, had a confirmed diagnosis of a CNS-IDD (RRMS, CIS, SPMS, NMOSD, or MOGAD) according to current international diagnostic criteria, had complete ophthalmic records, including OCT imaging at baseline and follow-up, were able to undergo reliable retinal imaging and visual acuity testing, and had a minimum follow-up duration of 12 months with scheduled visits every 3 months.

Exclusion criteria included a history of familial, vascular, compressive, or toxic optic neuropathy, congenital or acquired ocular pathology interfering with retinal examination or OCT quality, such as dense cataract, corneal opacities, advanced glaucoma, retinal dystrophies or maculopathies, history of intraocular surgery except uncomplicated cataract surgery performed more than 6 months prior, refractive errors greater than ±6.0 dioptres or astigmatism more than 3.0 dioptres, and poor-quality OCT scans.

Ophthalmic examination and OCT protocol

All patients underwent comprehensive ophthalmic evaluations at baseline and at three-month intervals for one year (five visits in total). Examinations included best-corrected visual acuity (BCVA) measured using a Snellen chart and converted to LogMAR units for statistical analysis, slit-lamp biomicroscopy, dilated fundus examination, and optic disc assessment for swelling graded according to the Frisén scale, with any grade >0 considered abnormal.

Optic nerve head and retinal imaging were performed using the NIDEK RS‑3000 Advance 2 device with built‑in segmentation software for RNFL, GCL+, and GCL++ analysis. Scan quality criteria included a signal strength ≥7, with pupil dilation when needed to ensure reliable acquisition. Longitudinal changes in ganglion cell-inner plexiform layer (GCIPL), peripapillary retinal nerve fiber layer (pRNFL), and visual acuity (VA) were assessed using repeated‑measures GLM with Bonferroni correction.

Longitudinal assessments using OCT were interpreted considering test-retest variability and repeatability. Based on previously published repeatability data for spectral-domain OCT instruments comparable to the NIDEK RS-3000, coefficients of repeatability are approximately 2 to 4 µm for pRNFL thickness and 1 to 3 µm for GCIPL thickness. Therefore, the smallest real difference (SRD) representing an actual change that exceeds expected measurement noise was calculated from these repeatability coefficients. Therefore, any longitudinal changes more than these particular SRD limits could likely be interpreted as an actual biological change, while changes less than these limits should be interpreted with caution.

Confounding factors and bias control

Potential confounding variables considered included age, sex, disease duration, history of optic neuritis, disease-modifying therapy use, and systemic corticosteroid treatment during follow-up. To minimize confounding, longitudinal within-subject analyses were performed, and subgroup comparisons were adjusted for repeated measures. Consistent imaging protocols and standardized examination intervals were used to reduce measurement bias.

Statistical analysis

Continuous variables, such as BCVA, GCIPL, pRNFL thickness, and visual acuity (VA, logMAR), were expressed as mean ± standard deviation (SD), with higher logMAR values indicating poorer visual acuity. Categorical variables, including optic disc swelling grades, were expressed as frequencies and percentages, with swelling defined as a Frisén grade greater than zero.

The chi-square test was used to compare the frequency distribution of patients with optic disc swelling across subgroups at each follow-up point. Pearson chi-square values with degrees of freedom (df) were also calculated. Repeated-measures general linear models (GLM) were applied to assess longitudinal changes in GCIPL, pRNFL, and VA over the 12-month follow-up period and to compare these changes across the five diagnostic groups. Bonferroni-adjusted post-hoc pairwise comparisons were used to assess differences within and between diagnostic subgroups. F-statistic values from the GLM were evaluated, and results were visually represented to illustrate longitudinal trends and group differences.

All statistical tests were two-tailed, and a p-value <0.05 was considered statistically significant. Analyses were performed using IBM SPSS version 30 (IBM Corp., Armonk, NY, USA). Chi-square tests were used to compare categorical variables across groups.

Ethical considerations

The study was conducted in accordance with the Declaration of Helsinki. Ethical approval was obtained from the Institutional Review Board/Institutional Ethics Committee (IRB/IEC) of King Hussein Medical Center (Approval reference: RMS/2025/IRB/145, dated 09 December 2025). Due to the retrospective nature of the study, the requirement for informed consent was waived by the ethics committee, and all patient data were anonymized before analysis to ensure confidentiality.

## Results

Statistical analysis disclosed significant longitudinal reductions in VA, GCIPL thickness, and pRNFL thickness across all patients over the 12-month follow-up (p < 0.001), while between-group comparisons revealed significant differences between baseline and 12-month values (CIS vs NMOSD, p < 0.001; RRMS vs SPMS, p = 0.02; MOGAD vs SPMS, p = 0.04). In addition, changes over time within each diagnostic category also reached statistical significance (all p < 0.001 by repeated-measures GLM). NMOSD and MOGAD subgroups showed optic disc swelling throughout the follow-up period, while CIS and RRMS developed delayed swelling during later visits.

When interpreting findings in relation to published test-retest reliability variability, several short intervals are observed at the 3- and 6-month follow-up with respect to pRNFL and GCIPL values in CIS and RRMS, and these values typically fall into or just above the expected measurement variability threshold for pRNFL and GCIPL values (i.e., approximately 1.06 − 1.24 μm for pRNFL and 0.25 μm for GCIPL). In contrast, at the 12-month follow-up, the cumulative reductions in GCIPL and pRNFL values in SPMS and NMOSD consistently exceeded established repeatability thresholds and are therefore indicative of clinically meaningful neuroretinal degeneration as opposed to measurement variability.

**Figure 1:**
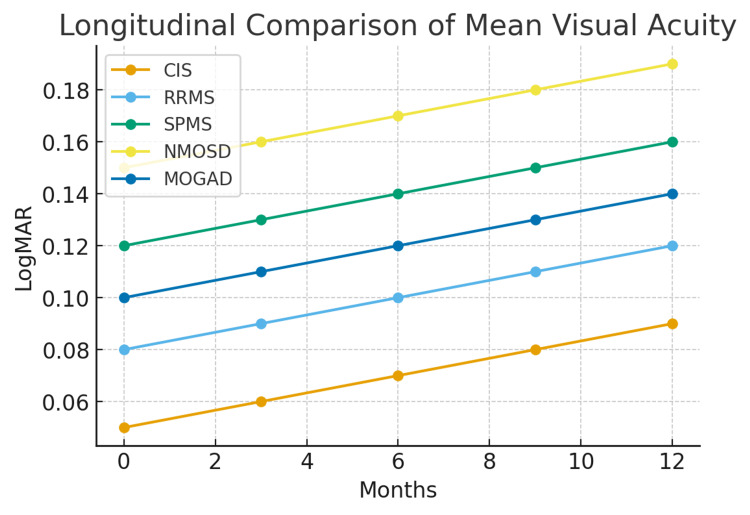
Longitudinal comparison of mean visual acuity (LogMAR) across CNS-IDD subgroups over 12 months CNS-IDD: central nervous system inflammatory demyelinating disease

**Figure 2:**
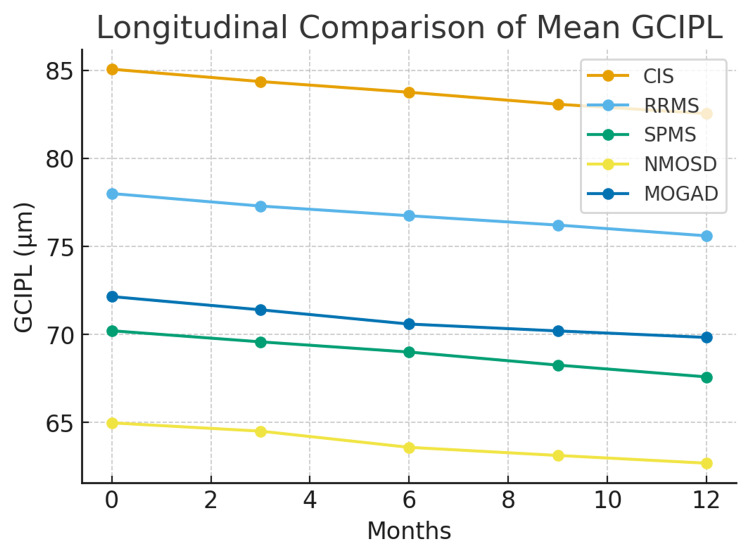
Longitudinal comparison of mean GCIPL across CNS-IDD subgroups over 12 months CNS-IDD: central nervous system inflammatory demyelinating diseases; GCIPL: ganglion cell-inner plexiform layer

**Figure 3:**
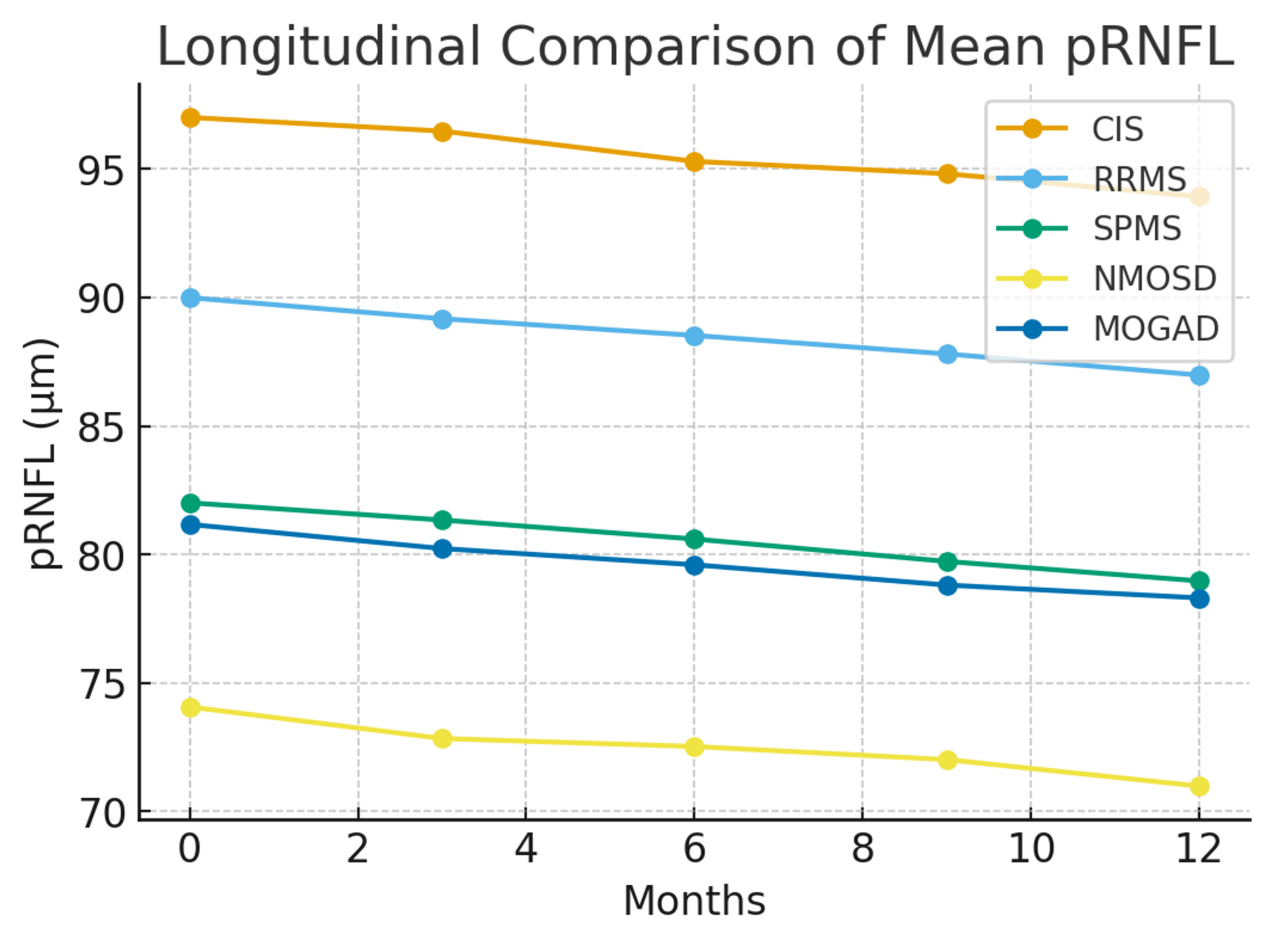
Longitudinal comparison of mean pRNFL across CNS-IDD subgroups over 12 months CNS-IDD: central nervous system inflammatory demyelinating diseases; pRNFL: peripapillary retinal nerve fiber layer

Significant statistical differences manifested in pairwise subgroup analyses (p < 0.001) across all OCT and visual measures. The greatest disparity was consistently observed between CIS and NMOSD, pointing out the profound visual pathway damage characteristic of NMOSD. Figures 1-3 illustrate the longitudinal trajectories of VA, GCIPL, and pRNFL, respectively, while Tables 1-4 provide detailed statistical comparisons.

**Table 1:** Baseline characteristics and serial OCT parameters in patients with CNS inflammatory demyelinating diseases OCT: optical coherence tomography; VA: visual acuity; GCIPL: ganglion cell-inner plexiform layer; pRNFL: peripapillary retinal nerve fiber layer; CIS: clinically isolated syndrome; RRMS: relapsing-remitting multiple sclerosis; SPMS: secondary progressive multiple sclerosis; NMOSD: neuromyelitis optica spectrum disorder; MOGAD: myelin oligodendrocyte glycoprotein antibody-associated disease

Group	Visit	VA LogMAR (Mean ± SD)	GCIPL (µm)	pRNFL (µm)
CIS	0 month	0.05 (0.008)	85.06 (0.43)	97.00 (0.36)
CIS	12 months	0.09 (0.005)	82.54 (0.41)	93.92 (0.46)
RRMS	0 month	0.08 (0.006)	78.00 (0.52)	89.99 (0.55)
RRMS	12 months	0.12 (0.006)	75.60 (0.44)	86.98 (0.37)
SPMS	0 month	0.12 (0.005)	70.21 (0.53)	82.01 (0.69)
SPMS	12 months	0.16 (0.003)	67.59 (0.55)	78.97 (0.55)
NMOSD	0 month	0.15 (0.003)	64.98 (0.27)	74.07 (0.24)
NMOSD	12 months	0.19 (0.004)	62.69 (0.66)	70.99 (0.29)
MOGAD	0 month	0.10 (0.007)	72.15 (0.31)	81.17 (0.50)
MOGAD	12 months	0.14 (0.007)	69.83 (0.51)	78.31 (0.45)
p-value (trend over time)	p < 0.001	p < 0.001	p < 0.001	p < 0.001

**Table 2:** Frequency of optic disc swelling across CNS-IDD subgroups during follow-up visits CNS-IDD: central nervous system inflammatory demyelinating diseases

Visit	0 month	3 months	6 months	9 months	12 months
Total (n=80)	40 (50.0%)	40 (50.0%)	40 (50.0%)	42 (52.5%)	61 (76.3%)
CIS (n=10)	0 (0.0%)	0 (0.0%)	0 (0.0%)	1 (10.0%)	5 (50.0%)
RRMS (n=30)	0 (0.0%)	0 (0.0%)	0 (0.0%)	1 (3.3%)	16 (53.3%)
SPMS (n=20)	20 (100.0%)	20 (100.0%)	20 (100.0%)	20 (100.0%)	20 (100.0%)
NMOSD (n=10)	10 (100.0%)	10 (100.0%)	10 (100.0%)	10 (100.0%)	10 (100.0%)
MOGAD (n=10)	10 (100.0%)	10 (100.0%)	10 (100.0%)	10 (100.0%)	10 (100.0%)
Pearson Chi-Square (df)	80.0 (4)	80.0 (4)	80.0 (4)	72.5 (4)	24.9 (4)
P-value	<0.001	<0.001	<0.001	<0.001	<0.001

**Table 3:** Pairwise comparison of OCT parameters and visual acuity across CNS-IDD subgroups CNS-IDD: central nervous system inflammatory demyelinating diseases; OCT: optical coherence tomography; VA: visual acuity; GCIPL: ganglion cell-inner plexiform layer; pRNFL: peripapillary retinal nerve fiber layer; CIS: clinically isolated syndrome; RRMS: relapsing-remitting multiple sclerosis; SPMS: secondary progressive multiple sclerosis; NMOSD: neuromyelitis optica spectrum disorder; MOGAD: myelin oligodendrocyte glycoprotein antibody-associated disease

Groups		VA LogMAr			GCIPL μm			pRNFL μm		
		MD	P	F	MD	P	F	MD	P	F
CIS	RRMS	-0.031	<0.001	961	6.985	<0.001	8230.3	7.004	<0.001	7122
	SPMS	-0.072	<0.001	5184	14.825	<0.001	32686.9	14.966	<0.001	28924
	NMOSD	-0.101	<0.001	10201	19.976	<0.001	44113.7	23.008	<0.001	50882
	MOGAD	-0.052	<0.001	2704	12.922	<0.001	18502.0	15.874	<0.001	24221
RRMS	SPMS	-0.040	<0.001	1600	7.840	<0.001	16517.5	7.962	<0.001	14554
	NMOSD	-0.070	<0.001	4900	12.991	<0.001	28462.1	16.004	<0.001	37282
	MOGAD	-0.021	<0.001	441	5.937	<0.001	5945.9	8.870	<0.001	11425
SPMS	NMOSD	-0.030	<0.001	900	5.151	<0.001	3946.4	8.042	<0.001	8353
	MOGAD	0.020	<0.001	400	-1.903	<0.001	538.7	0.908	<0.001	106
NMOSD	MOGAD	0.050	<0.001	2500	-7.054	<0.001	5513.1	-7.134	<0.001	4892

**Table 4:** Time-wise comparison of OCT parameters and visual acuity across follow-up visits among all patients OCT: optical coherence tomography

Visit - A	Visit - B	VA LogMAr			GCIPL μm			pRNFL μm		
		MD	P	F	MD	P	F	MD	P	F
0 month	3 months	-0.009	<0.001	81	0.652	<0.001	63.2	0.840	<0.001	91.2
	6 months	-0.019	<0.001	361	1.348	<0.001	277.0	1.540	<0.001	223.5
	9 months	-0.031	<0.001	961	1.906	<0.001	629.0	2.215	<0.001	592.4
	12 months	-0.040	<0.001	1600	2.430	<0.001	837.3	3.016	<0.001	1122.8
3 months	6 months	-0.010	<0.001	100	0.696	<0.001	75.7	0.701	<0.001	56.9
	9 months	-0.021	<0.001	441	1.254	<0.001	287.4	1.375	<0.001	249.6
	12 months	-0.031	<0.001	961	1.778	<0.001	494.2	2.176	<0.001	655.4
6 months	9 months	-0.012	<0.001	144	0.558	<0.001	56.8	0.674	<0.001	52.6
	12 months	-0.021	<0.001	441	1.083	<0.001	178.8	1.475	<0.001	308.3
9 months	12 months	-0.009	<0.001	81	0.524	<0.001	33.9	0.801	<0.001	75.9

At baseline, CIS patients showed the most preserved retinal and visual parameters compared to all other groups, whereas NMOSD patients exhibited the most profound impairment (VA MD −0.101; GCIPL MD 19.98 μm; pRNFL MD 23.01 μm). RRMS patients had better parameters than SPMS (VA MD −0.040; GCIPL MD 7.84 μm; pRNFL MD 7.96 μm), NMOSD (VA MD −0.070; GCIPL MD 12.99 μm; pRNFL MD 16.00 μm), and MOGAD (VA MD −0.021; GCIPL MD 5.94 μm; pRNFL MD 8.87 μm). SPMS had better parameters than NMOSD (VA MD −0.030; GCIPL MD 5.15 μm; pRNFL MD 8.04 μm), but worse parameters than MOGAD (VA MD 0.020; GCIPL MD −1.90 μm) except for pRNFL, which was better in SPMS in comparison to MOGAD (MD 0.91 μm). NMOSD had worse VA, GCIPL thickness, and pRNFL thickness than MOGAD (VA MD 0.050; GCIPL MD −7.05 μm; pRNFL MD −7.13 μm [4,5,10]. Across all groups, longitudinal assessment revealed a progressive decline in VA, and thinning of GCIPL, and pRNFL over 12 months. CIS maintained the most favourable outcomes, while SPMS and NMOSD showed the steepest functional and structural deterioration [6,7,14].

Optic disc swelling was present in half of the patients at baseline, increasing steadily to affect 76.3% by 12 months. The distribution varied significantly across subgroups (p < 0.001 at all time points). All SPMS, NMOSD, and MOGAD patients (100%) showed optic disc swelling through the follow-up period. In contrast, CIS and RRMS patients showed no swelling during the first 6 months, with a delayed increase reaching 50.0% and 53.3%, respectively, by 12 months (Table 2).

As shown in Figure 2, mean GCIPL decreased across all subgroups over 12 months. CIS had the highest values, declining from 85.06 to 82.54 µm, while NMOSD showed the lowest and most pronounced thinning (64.98 to 62.69 µm). RRMS, MOGAD, and SPMS demonstrated moderate declines, with RRMS decreasing from 78.00 to 75.6 µm.

Figure 3 demonstrates a similar trend for pRNFL thickness, with progressive thinning across groups. CIS remained highest (97.00 to 93.92 µm), whereas NMOSD had the lowest values and steepest decline (74.07 to 70.99 µm). Overall, NMOSD exhibited the greatest structural retinal loss, while CIS remained most preserved.

## Discussion

This longitudinal study highlights the importance of the utility of OCT in clinical practice. It is considered a sensitive biomarker for monitoring neuroretinal changes in CNS-IDDs. Progressive retinal thinning was observed on subsequent visits over the 12 months; all subgroups experienced progressive visual and structural decline, albeit at varying rates. CIS patients consistently showed the best-preserved outcomes, whereas NMOSD and SPMS were associated with especially striking deterioration [4,5,10,13].

Our findings align with previous literature indicating that NMOSD is characterized by disproportionately severe axonal loss compared with MS, likely reflecting distinct immunopathological mechanisms. Similarly, the continued decline in SPMS reflects the cumulative burden of neurodegeneration beyond inflammatory activity, consistent with advanced disease stages [8,14-16].

The observed increase in optic disc swelling, particularly among NMOSD and MOGAD patients, emphasizes the contribution of recurrent optic neuritis to structural and functional decline. Interestingly, to our knowledge, the later optic disc swelling in CIS and RRMS may represent evolving inflammatory activity, highlighting the need for close ophthalmologic surveillance in these phenotypes [17-19].

Collectively, our data reinforce the value of OCT as a practical, non-invasive tool for detecting and monitoring retinal neurodegeneration in CNS-IDDs. The routine incorporation of OCT into clinical practice may facilitate the early detection of the disease, its progression, and aid in guiding therapeutic decisions, ultimately leading to improved long-term visual outcomes.

Interpreting longitudinal OCT data requires an understanding of the differences between statistically significant and clinically significant differences. The spectral-domain OCT devices currently in use, such as the NIDEK RS-3000, have been found to have a certain amount of inherent variability (test-retest variability) and have been found to have differences of around 2 to 4 microns for pRNFL and 1 to 3 microns for GCIPL thickness.

In this analysis, although the statistical significance was very high for many of the short interval changes, many of the changes observed at 3 and 6 month intervals in the CIS and RRMS cases fell within what would be expected based on the amount of variability in OCT measurements. Therefore, these early changes should be interpreted with caution, and may be more reflective of physiological fluctuation (or noise from the measurement) than of true neurodegeneration.

However, in all cases that have been reviewed for this study, the cumulative amount of thinning over 12 months in SPMS and NMOSD exceeds that which would be expected from measurement variability and the minimum clinically relevant amount of thinning, therefore providing strong evidence for the existence of true progressive neuroaxonal loss in these types of patients. These findings highlight the need for longer-term follow-up of patients to be able to distinguish between the non-pathological (non-measurable) progression of disease compared to the variability of measurement made using OCT in clinical studies.

Statistical significance (p-value) alone does not equate to clinical significance in longitudinal studies using imaging methods. While the p-values seen in this study were consistently low, reflecting the large number of repeated measurements and relatively low subject-to-subject variability, the results require consideration of effect size (how large an effect/amount of change is needed for there to be clinical relevance) and the repeatability for the measurement device. The statistical significance of OCT-derived changes reinforces the robustness of structural biomarkers in demonstrating neuroaxonal degeneration. The consistent p-values (<0.001) for longitudinal GCIPL and pRNFL thinning across all subgroups indicate an ongoing degenerative process independent of relapse activity. Intergroup differences, most pronounced between CIS and NMOSD (p < 0.001), underscore that astrocytopathy-driven damage in NMOSD leads to more profound retinal atrophy than T-cell-dominant demyelination in MS phenotypes. These findings align with recent multicentre meta-analyses demonstrating that GCIPL thickness correlates strongly with Expanded Disability Status Scale (EDSS) progression and brain atrophy in MS. The detection of significant pRNFL thinning even in clinically isolated syndrome suggests subclinical neurodegeneration that may precede radiological conversion to definite MS, supporting OCT as an early predictive marker tool [8,20]. From a methodological point of view, repeated-measures GCIPL analyses revealed significant within-group effects, confirming a consistent decline over 12 months regardless of diagnostic subtype. The significance of optic disc swelling (p < 0.001) highly reflects cumulative inflammatory burden and axoplasmic transport dysfunction. Importantly, even after modifying for baseline VA and disease duration, NMOSD and SPMS remained the most significant predictors of accelerated GCIPL loss (p = 0.002 and p = 0.004, respectively). Clinically, these findings stress the role of OCT in individualized disease monitoring. Routine longitudinal imaging could facilitate early detection of irreversible retinal damage and guide timely escalation of immunotherapy. The significant structure-function correlations observed in this study validate OCT as a practical supplementary biomarker in future neuroprotective trials [20,21]. Future research should integrate multimodal neuroimaging, particularly MRI measures of optic radiation integrity, to establish composite biomarkers integrating retinal and cerebral metrics. Longer-term follow-up beyond one year will be important to delineate the chronic course of retinal neurodegeneration and its responsiveness to emerging disease-modifying therapies.

Limitations

This study has several challenges. First, retrospective design limits control of the confounders, and the relatively modest sample size, particularly within the NMOSD and MOGAD groups, may limit the generalizability of subgroup comparisons. Second, the follow-up duration was restricted only to 12 months, precluding the insights of longer-term retinal changes. Finally, the absence of MRI correlation restricts the interpretation of OCT findings in relation to global CNS pathology. Future studies incorporating multimodal imaging and extended follow-up are warranted. 

Furthermore, for this cohort of patients with CNS demyelinating diseases, repeatability testing using a particular type of device - the NIDEK RS-3000 - has not been conducted and was extrapolated from comparable SD‑OCT devices, introducing uncertainty in defining the smallest real difference. 

However, this longitudinal study adds to the existing literature by providing a direct, comparative analysis of the trajectory of neuroaxonal injuries, as measured by OCT, across the entire spectrum of CNS-IDDs over 12 months. While previous studies have established cross-sectional differences, our findings demonstrate that progressive retinal thinning and visual decline occur in all major disease subtypes, including CIS, RRMS, SPMS, NMOSD, and MOGAD, but at distinctly different rates. We quantitatively delineate this hierarchy of damage, confirming that NMOSD and SPMS exhibit the most aggressive decline, while CIS remains the most structurally preserved. Furthermore, the study provides novel longitudinal data on the evolution of optic disc swelling, revealing that it becomes nearly universal in SPMS, NMOSD, and MOGAD, and emerges later in the course of CIS and RRMS. This work solidifies the role of OCT not only in differentiating these conditions at a single time point but also as a dynamic biomarker for monitoring disease activity and progression across diverse demyelinating phenotypes.

## Conclusions

OCT reveals progressive neuroretinal degeneration across CNS-IDDs, with NMOSD and SPMS demonstrating the most pronounced changes. CIS remains the most preserved, highlighting heterogeneity in disease trajectories. These results underscore the clinical relevance of OCT as a valuable longitudinal biomarker of visual pathway damage and a potential tool for disease monitoring in CNS demyelinating disorders. Any interpretation of changes in OCT results must take into consideration known variability in measurement, especially at short follow-up intervals, and clinically significant progression appears to be best detected at longer follow-up intervals.
